# How predictable is adaptation from standing genetic variation? Experimental evolution in *Drosophila* highlights the central role of redundancy and linkage disequilibrium

**DOI:** 10.1098/rstb.2022.0046

**Published:** 2023-05-22

**Authors:** Christian Schlötterer

**Affiliations:** Institut für Populationsgenetik, Vetmeduni Vienna, Veterinärplatz 1, 1210 Wien, Austria

**Keywords:** experimental evolution, parallel evolution, predicting evolution, laboratory natural selection, truncating selection, adaptive architecture

## Abstract

Experimental evolution is well-suited to test the predictability of evolution without the confounding effects of inaccurate forecasts about future environments. Most of the literature about parallel (and thus predictable) evolution has been carried out in asexual microorganisms, which adapt by de novo mutations. Nevertheless, parallel evolution has also been studied in sexual species at the genomic level. Here, I review the evidence for parallel evolution in *Drosophila*, the best-studied obligatory outcrossing model for adaptation from standing genetic variation in the laboratory. Similar to asexual microorganisms, evidence for parallel evolution varies between the focal hierarchical levels. Selected phenotypes consistently respond in a very predicable way, but the underlying allele frequency changes are much less predictable. The most important insight is that the predictability of the genomic selection response for polygenic traits depends highly on the founder population and to a much lesser extent on the selection regime. This implies that predicting adaptive genomic response is challenging and requires a good understanding of the adaptive architecture (including linkage disequilibrium) in the ancestral populations.

This article is part of the theme issue ‘Interdisciplinary approaches to predicting evolutionary biology’.

## 1. Background

Natural populations are constantly facing changes in their environment. In light of the current rate of global climate change, however, it is particularly important to predict how the flora and fauna with their extended genomes will respond. Apart from the challenge to generate correct models of climate change, it is not clear to what extent we are able to predict the selective response based on the past. Some progress has been made towards predicting, for example influenza outbreaks [1], or the fitness of genotypes in different environments [2]. Nevertheless, even phenotypic changes that are apparently connected to environmental change, such as the beaks of Darwin's finches, are not very predictable [3]. A simple explanation for the low predictability of adaptive responses comes from the difficulty of capturing relevant ecological parameters from the past to make predictions about the future [4].

An alternative, although less comprehensive, approach is the analysis of replicate populations exposed to similar environments. Enigmatic examples of parallel adaptation include the parallel armour plate reduction in independently colonized freshwater populations of the three-spine stickleback [5], crypsis in beach mice [6] and adaptation to the myxoma virus in rabbit populations in different countries [7]. The adaptation of human populations to low oxygen partial pressure at high altitudes is another classic example of the ability of natural populations to adapt to new environments. Unlike in sticklebacks, where the same allele of the Eda locus is responsible for the parallel response, human populations adapted by changes at different, but partially overlapping sets of genes [8–10]. The contrast of these two cases of parallel adaptation already shows the limitation of studying parallel adaptation in natural populations. It remains unclear whether the differences among replicates reflect allele frequency differences—including the absence or fixation of adaptive alleles—or environmental heterogeneity. Indeed, many examples of genetic heterogeneity (i.e. different contributing loci) are known within and between species [11–13].

Experimental evolution, on the other hand, provides a unique opportunity to control for environmental uncertainty and to gain insight into the parallel nature of adaptation. Given the ease of maintaining large population sizes and short generation times, asexual microorganisms have been the work horse of studies of parallel evolution (reviewed in [14]). Because these experiments start from a single genetically identical clone, populations must adapt by de novo mutations. In a particularly highly replicated experiment, 115 replicate *Escherichia coli* populations were exposed to a novel hot temperature regime [15]. The important insight was that the predictability of evolution depends on the focal hierarchical level. Pathways related to RNA polymerase complexes or the termination factor *rho* were modified in multiple replicates. The parallel changes in pathways were achieved by mutations in different genes and almost no parallelism was detected on the level of individual mutations [15]. The variable degree of parallelism at different hierarchical levels shows that adaptation to a novel environment can be achieved by mutations in different sets of genes, which are nevertheless contributing to similar pathways. The phenomenon that only a subset of all these loci is needed to achieve the selected response has been called genetic redundancy [16,17]. Other studies indicated that for certain traits, such as antibiotic resistance, only a limited number of mutational paths are accessible because resistance mutations interact epistatically. Such epistatic interactions between adaptive single nucleotide polymorphisms (SNPs) facilitate parallel evolution, not only on the pathway level, but also for individual SNPs [18].

Unlike microorganisms, experimental evolution in sexual species typically does not rely on de novo mutations but takes advantage of standing genetic variation [19]. Adaptation from standing genetic variation is expected to result in more parallel adaptation than adaptation from de novo mutations because the stochasticity of the mutational process is bypassed. On the other hand, epistasis may not have the same importance in sexual organisms as in asexual ones. First, harmful epistatic mutations may be purged, and second, recombination continuously breaks down epistatic associations. Empirical support for this expectation is difficult to obtain because experiments from standing genetic variation are problematic in asexual microorganisms. Furthermore, the population sizes of sexual organisms are too small to test parallel evolution from de novo mutations. Nevertheless, replicated experiments starting from the same founder population are highly informative about the upper bound of parallel evolution from standing genetic variation.

*Drosophila* provides an excellent study system to investigate parallel adaptation from standing genetic variation with recombination. The combination of experimental evolution with whole genome re-sequencing of pools of individuals (Pool-Seq [20]) permits the monitoring of genome-wide allele frequency changes in multiple replicates at different time points [19,21]. Such allele frequency trajectories provide unprecedented insights into the dynamics of adaptive alleles and their similarity across replicate populations.

## 2. Convergent evolution

Some authors distinguish between parallel and convergent evolution, where convergent evolution refers to adaptation of genetically diverged populations or different species. Parallel evolution, however, starts from genetically highly similar founder populations and changes in the same direction (i.e. evolution from replicated founder populations). The potential of convergent evolution has been studied in a range of *Drosophila* species using genetically diverged founder populations from different locations. *Drosophila pseudoobscura* populations from five different locations were selected for ethanol knockdown resistance and converged to similar knockdown phenotypes within 18 generations of truncating selection [22]. Ethanol and acetone tolerance also evolved, but differed in magnitude between the populations, suggesting that they converged for the selected trait, but achieved this with different mechanisms. In a similar experiment with *Drosophila melanogaster,* clinal populations evolved towards a higher ethanol knockdown resistance, but did not converge to the same trait value [23]. Another interesting case of convergent phenotypic evolution was observed in *Drosophila subobscura*. Three founder populations with different geographical origins adapted to laboratory culture conditions. For a range of morphological, physiological and life-history traits, convergent evolution to the same adaptive state was observed, although different evolutionary paths were used [24]. Nevertheless, when a larger set of *D. subobscura* populations was studied, the extent of convergence differed between traits [25]. For two populations with fast phenotypic convergence, the genomic selection response was analysed by Pool-Seq and no convergence was detected between populations for selected SNPs after 50 generations in the laboratory [26]. Consistent with high genetic redundancy, this result confirms that alternate adaptive paths were used in the different populations.

Overall, these experiments suggest that convergent evolution is a very likely outcome for selected phenotypes, but on the genomic level different starting frequencies of contributing loci may have led to the use of alternate paths of adaption, which nevertheless resulted in convergent evolution for the selected trait(s).

## 3. Variable levels of parallelism at different hierarchies

The limited convergence of genetically diverged populations on the genetic level reflects genetic redundancy, heterogeneous effect sizes and different starting frequencies of contributing loci. For experiments starting with a similar genetic composition the selection response among replicates is expected to be more parallel and thus more predictable. This hypothesis can be scrutinized by the evolutionary response of replicate populations starting from the same founder population. Griffin *et al*. [27] used freshly established isofemale lines to generate 10 genetically highly similar replicate populations. Half the population were control lines evolving to laboratory conditions. The other half was exposed for 13 generations to directional selection for increased desiccation resistance and showed a very pronounced change for the selected phenotype. No significant phenotypic difference was observed among the five replicates, suggesting a high level of parallelism for the selected trait. On the genomic level, different sets of SNPs responded to desiccation resistance in each of the five selected replicates. A low level of parallelism on the genomic level was also seen for laboratory adaptation in the control lines, although the selection response was much weaker [27].

Unlike [27], Barghi *et al*. [28] did not select for a specific trait. They exposed freshly collected fly cultures from Florida to a hot temperature regime in the laboratory—an approach called laboratory natural selection [29] because no specific trait is being selected by the experimenter. Rather, fitness differences between the genotypes segregating in the population are driving adaptation to the novel environment—similar to natural selection, but under controlled laboratory conditions. After 60 generations the evolved flies showed higher fitness (fecundity) compared to the ancestral population and also evolved changes in lipid content and CO_2_ production [28]. Since all replicate populations changed their phenotypes in the same direction, the evolution of all measured high-level phenotypes was parallel, although it is not clear to what extent they are direct targets of selection. Interestingly, while the differences in fecundity among the evolved replicates were not significant [28], a later study showed that the relative differences among evolved populations were maintained for at least 20 generations. Hence, for this fitness component the evolved replicates responded in the same direction, but the phenotypic response did not fully converge to the same trait value [30]. On the genomic level, the differences among replicates were even more pronounced. In total 99 selected haplotype blocks were identified, which increased more in frequency than expected under a pure genetic drift model [28]. Only a single selected haplotype block was shared among all 10 replicates and on average a given haplotype block was selected only in five replicates. Given the moderate effective population sizes in the experiment, some heterogeneity among replicates can be caused by genetic drift even when each selection target is contributing independently of the other selection targets (selective sweep model). Nevertheless, computer simulations showed that the low level of parallel response is much better explained by genetic redundancy where different combinations of loci (selected haplotype blocks) contribute to the observed change in high-level phenotypes. These results have important implications for our expectations of parallel evolution: if the required phenotypic changes can be obtained by different combinations of genes, parallel phenotypic changes can be driven by non-parallel genetic changes—even when replicate founder populations are genetically highly similar.

A gene expression analysis of the same evolution experiment further illustrates the importance of the hierarchical level at which the parallelism of selection responses is determined. Gene expression was measured in the ancestral population and two evolved replicates [31]. For 125 genes in the first replicate and 97 genes in the second replicate the variance in gene expression was significantly reduced in the evolved replicates compared to ancestral populations. Only 11 genes showed a significant reduction in variance in both replicates [31]. Although this overlap is more than expected by chance, the small number of genes with a similar expression variance change between replicates is consistent with the low parallelism observed on the DNA level. In both replicates the significant genes were enriched for the same gene ontology (GO) categories and in the same tissue type (midgut) [31]. This indicates that even without a parallel response at the level of individual genes, the populations experienced a similar functional shift during adaptation. Nevertheless, the low parallelism of the expression evolution does not apply to all genes. In the same experiment highly parallel gene expression changes were also observed. For example, genes involved in dopamine signalling evolved in a highly parallel way, such that even in whole-body transcriptomic analysis gene expression changes restricted to a small number of neuronal cells could be detected [32].

Hence, overall, replicate populations evolving from the same founder populations showed contrasting patterns of parallelism, depending on the hierarchical level studied. Phenotypes selected by experimental design or high-level phenotypes, such as fecundity, CO_2_ production and metabolism with a close connection to the selected trait(s) responded in a highly parallel way, but on the genomic and to some extent also on the transcriptomic level the response was considerably more heterogeneous, resulting in a low level of predictability.

## 4. The degree of parallelism depends on the founder population, not on the selection regime

Mallard *et al*. [33] used the same selection regime and experimental set-up as in [28], but the founder population originated from Portugal rather than Florida [34]. Consistent with the Florida experiment, the phenotypic response was highly parallel across replicates. In this Portugal study, however, the genomic response was highly parallel with a small number of strongly selected loci [34]. Furthermore, the gene expression changes were also highly parallel in this population [33]. This different behaviour among replicates from the two different founder populations is particularly striking as they were subjected to the same selection regime in the same laboratory and raises the question of why predictability of adaptation differs between the two experiments.

In an attempt to shed more light on this different behaviour, replicate populations from the same Portuguese founder population were studied for approximately the same number of generations in a cold selection regime (10–20°C) [35]. Interestingly, the genomic response at the cold temperature was also highly parallel, similar to the hot temperature regime [28]. It was concluded that parallelism depends more on the nature of the founder population, than on the actual selection regime. The Florida and Portugal founder populations differed in two salient features. The Portugal population was less polymorphic and exhibited more linkage disequilibrium (LD) than the Florida founder population. Both factors could explain the differences in parallel selection responses between the two populations, but the authors favoured LD as a more likely explanation because the differences in variability were not very large [35].

## 5. Highly parallel genomic responses from laboratory adapted populations

Many evolution studies in *Drosophila* use laboratory-adapted populations to ensure that the response to focal selection regimes is not confounded by adaptation to laboratory conditions. Using the comparison of replicate populations evolved under an accelerated development regime for 600 generations, Burke *et al*. [36] inferred highly parallel phenotypic selection responses. The evidence for a highly parallel genomic selection response was initially based only on the contrast of a single replicate to pooled replicates [36], but it was later confirmed by sequencing all evolved replicates [37]. Another example for highly parallel selection responses from laboratory adapted founder populations comes from three selection regimes differing by the age of reproduction (one regime was shared with [36]). The interesting aspect of this study is that the selection regimes were started at two different time points [38]. Unlike yeast, *E coli* and *Caenorhabditis elegans, Drosophila* cannot be frozen, thus selection experiments starting at two different time points cannot rely on the same founder population. Graves *et al*. [38] solved this challenge by using a founder population which was derived from the same ancestral population used in the first time point. Furthermore, not all selection regimes of the first time point used the same founder population. As a consequence, founder populations from the first and second time point were probably genetically distinct, either by selection or drift. Nevertheless, the authors observed a remarkable parallel response on the phenotypic and genomic level [38]. Not a single SNP was significantly differentiated between the experiments that started at different time points for each of the three selection regimes. Yet, contrasts between selection regimes consistently identified differentiated SNPs, confirming that all populations responded to selection.

The results of these two studies are very difficult to reconcile with a polygenic architecture of the targeted traits where more genetic variation was segregating in the founder populations than required for the adaptive response (genetic redundancy). Under this assumption, some differences in selection response would have been expected—simply owing to different starting frequencies of the contributing alleles in the two founder populations. This apparent discrepancy has been addressed by computer simulations, which confirmed that a high parallel genetic response is unexpected, given the estimated population sizes and a moderate complexity of the trait [37]. The authors suggested that migration between replicates may explain the low differentiation, but it is not clear if migration could also have occurred between the replicates from the two different time points in [38]. A hitherto unexplored explanation for this surprising parallelism is persistent LD such that selection causes frequency shifts of large haplotype blocks. It is, however, not apparent how LD could persist across many generations. Inversions can be ruled out because the X-chromosome is free of inversions, but follows the same pattern of parallel evolution [38]. It is not clear whether epistasis or genetic incompatibilities can be sufficiently frequent and strong enough to suppress recombination to the required extent. Some insights into the importance of LD in these experiments comes from SNP genotyping of populations that evolved for 50 generations during which the age of reproduction was reversed (reverse selection). Interestingly, not only the phenotypes reversed to the ancestral state, but also randomly selected SNPs did so in a very consistent way across replicates [39]. Since it is unlikely that these SNPs were direct targets of selection, this study highlights the importance of LD in these studies. More genomic analyses from different laboratory adapted populations are needed to confirm the generality of highly parallel selection responses with this experimental setting.

## 6. Highly parallel genomic selection responses with two founder genotypes

The importance of LD for parallel genomic selection signatures can be demonstrated by the cross between two inbred lines—a very extreme design for the genomic response to a new environment. Ten replicate populations from a cross between two inbred *D. melanogaster* isofemale lines were exposed to the extreme temperature of 29°C. After 20 generations, very strong and extremely parallel allele frequency changes were observed [40]. Hence, unlike the pattern seen for experiments with a complex founder population, the combination of LD and equal, high starting frequencies across the entire genome in a two-genotype experimental set-up allows for highly parallel selection responses—even with moderate population sizes.

## 7. Simple genetic architectures result in parallel evolution

In all studies discussed up to now, adaptation was complex and involved many contributing loci—more than needed to change the selected phenotype. An intrinsic property of this genetic redundancy is that parallel selection responses are expected for high-level phenotypes associated with the selected phenotype even when different loci are mediating this phenotypic shift [17]. Nevertheless, a very parallel selection response is expected for traits with a simple genetic basis. One example for such a simple trait is resistance to *Drosophila* C virus (DCV). Exposing replicates established from a freshly collected *D. melanogaster* population to DCV for 20 generations identified two selection targets, which responded in a highly parallel fashion across replicates and explained the parallel phenotypic response [41].

## 8. Conclusion

### (a) Redundancy and hierarchical levels

Experimental evolution with replicated populations adapting under the same selection regime provides a great opportunity to study the genetic components of predictability of evolution because the experimental design eliminates the challenge to predict environmental change. Phenotypes that are direct targets of selection, such as DCV resistance, desiccation resistance or ethanol knockdown resistance, respond in a highly predictable way. Remarkably, it does not matter whether the founder populations are genetically homogeneous or differentiated (questioning the distinction between parallel and convergent evolution). A similarly parallel selection response was seen for high-level phenotypes in laboratory natural selection experiments, in which selection pressure is generated by an environmental shift. Fitness not only increased in a very predictable way, but changes in CO_2_ production and lipid content were also highly parallel. Even GO categories were enriched for genes with a significant evolution of gene expression. Hence, these predictable changes provide a reliable indicator that these traits contribute to the observed fitness increase.

Gene expression has an intermediate position in the hierarchy—lower than high-level phenotypes, but higher than genomic variation. Even within the same experiment, gene expression exhibits variable levels of parallel response—while some genes respond in a highly parallel fashion, others differ among replicates. The heterogeneous degree of parallel selection response for gene expression may be explained by a range of factors, such as level of pleiotropy, strength of selection, regulatory architecture and thus ultimately redundancy.

Genomic changes are generally less parallel than the phenotypic response of complex traits in experimental evolution studies starting from freshly collected founder populations. Even when the founder populations are genetically very similar and all alleles are present in all replicates at almost identical frequencies, alternative combinations of loci contribute to parallel evolution of high-level phenotypes.

The dependence of parallelism on the hierarchical level studied is a direct consequence of redundancy. On the genomic level, many different variants are segregating in natural populations which can contribute to adaptation. In combination with variation in starting frequencies and genetic drift, replicate populations pursue different paths of adaptation. With increasing levels of the trait hierarchy, the selection response becomes more parallel because fewer alternative paths are available. Most predictable are the responses of selected phenotypes, which always respond in a parallel way.

The dependence of parallel evolution on the hierarchical level is very similar for adaptation from standing genetic variation and de novo mutations [14]. In both cases non-parallel evolution is the consequence of stochastic forces. In experiments using de novo mutations for adaptation, the stochastic factor is the timing of mutational events; for standing genetic variation genetic drift causes heterogeneity among replicates. Computer simulations show that with genetic redundancy the adaptive response from standing genetic variation is more predictable in large populations than in small ones [42]. This higher predictability in large populations reflects the reduced loss of contributing alleles by genetic drift and the improved discrimination of alleles with differences in effect size. Some empirical support for parallel allele frequencies in large populations comes from experiments in semi-natural enclosures with very large census sizes—much larger than for typical experimental evolution studies in the laboratory [43]. Although the observed allele frequency changes were small and changed direction during the experiment, parallel changes across replicates were detected [43].

### (b) Linkage disequilibrium

The reviewed studies ranged from almost perfect repeatability to very low repeatability. One simple explanation for the observed differences between experiments is the complexity of the selected trait. DCV resistance has a simple genetic basis with a small number of large effect alleles, which results in highly parallel selection responses. Nevertheless, other studies which selected for presumably complex traits still found very different levels of parallel response. Please note that a comparison of parallel responses across experiments does not ask whether the same genes/genomic regions are responding between experiments, but how the degree of parallelism between replicate populations of the same selection regime/founder population compares to other experiments. Hence, very different genes may respond to selection in two experiments, but the pattern of parallelism may still be very similar.

**Figure 1.**
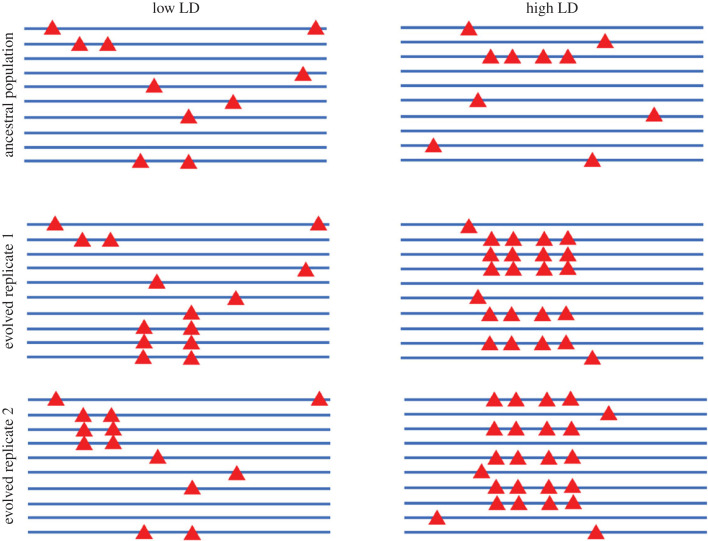
Schematic illustration of the impact of LD on selection response. Two founder populations with the same number of beneficial alleles (red triangles) are shown. In the left founder population the beneficial alleles are in low LD, in the right founder population four beneficial alleles occur on the same chromosome. The linked beneficial alleles are more strongly selected, resulting in a parallel pronounced frequency increase of this chromosome in both replicate populations. In the population with low LD a weaker, heterogeneous selection response is observed. (Online version in colour.)

A thought-provoking result was that parallel evolution on the sequence level depends strongly on the founder population. The same selection regime applied to diverged founder populations resulted in different levels of parallelism among the replicates of each founder population [35]. In the light of this observation, it is remarkable the same founder population exposed to two different selection regimes [35,38] resulted in a very similar levels of parallelism (i.e. the parallelism among replicates was similar for each selection regime, but not parallel for both regimes). Hence, for the level of parallel response among replicate populations, the selection regime seems to be of less importance than the founder population. The interpretation of these results is not straightforward. Computer simulations with a restricted set of parameters could only explain the high levels of parallel selection response observed in [36] by relatively high rates of gene flow [37]. Otte *et al*. [35] proposed an alternative explanation for different levels of parallel selection responses—LD in combination with genetic redundancy. When many loci are contributing to adaptation, a population with high LD will respond in a more predictable way than a population with low LD. The reason for more parallel selection response with high LD is the stochastic local accumulation of contributing alleles with effects in the same direction. This will result in a haplotype block with a large net effect (i.e. the sum of all contributing alleles in this haplotype block). The selective response of such a haplotype block will be strong and highly parallel (figure 1). This effect of linkage and clustering of alleles with effects in the same direction has also been shown in a theoretical study where such a haplotype block that contained many loci with aligned effect sizes introgressed into a population in a highly parallel way [44].

It is apparent that more data are required to further substantiate the role of LD for parallel evolution, but if this is a widespread phenomenon it has far-reaching implications for our understanding and interpretation of parallel evolution. First, it will not be sufficient to know the number of contributing loci and their effect sizes. The linkage structure in a population will also need to be accounted for to make predictions about the expected adaptive response on the sequence, but not the phenotypic, level. Second, even strong, highly parallel selection responses may not be the outcome of a single, strongly selected locus, but the joint contribution of many small effect loci in LD. This will make the functional validation of selected alleles a daunting task. Third, the adaptive response will be predictable for only a small fraction of the genome (where alleles with aligned effects are clustered); the dynamics of other contributing alleles remain largely stochastic.

Although many different selection regimes have been applied to polymorphic *Drosophila* populations, the evolutionary response for most of them is consistent with a complex genetic basis and the rare occurrence of large effect alleles. Nevertheless, strong genomic selection responses are detected in all studies, which is best explained by clustering of aligned small effect alleles on a single haplotype block [44,45]. The moderate population sizes used in current experimental evolution studies exploit genetic drift to uncover alternative genetic paths, which can be followed to achieve the same phenotypic response. More LD in the founder population will cause more parallel selection responses and genetic redundancy will be less apparent. Furthermore, larger effective population sizes could be used to uncover differences in effect sizes between contributing haplotype blocks. It is anticipated that the next generation of experimental evolution studies will take advantage of these insights and use innovative experimental designs to characterize the adaptive architecture [17] at a resolution which will be impossible in natural populations. Hence, while genome-wide selection scans of natural populations are expected to detect large effect alleles or clustered alleles of aligned small effects [46], experimental evolution can complement the analysis of natural populations and genome-wide association studies to obtain a more complete understanding of adaptive architecture (box 1).

Box 1. *Detecting non-parallel responses*

One interesting approach to study parallel evolution considers a multidimensional trait space by asking whether the vector connecting the centroids of the ancestral and evolved population is parallel and of similar length in other pairs of ancestral and evolved populations [47]. In this review I use a much more restricted definition of parallel evolution by focusing on an univariate character which changes its value in the evolved population relative to the ancestor.

*DNA sequence polymorphisms*

Population genetics provides an elegant theoretical framework to describe the expected allele frequency changes under neutrality [48]. This provides a powerful machinery to determine whether the observed allele frequency changes in a single replicate are more extreme than expected under neutrality. If the same locus displays a significant allele frequency change in the same direction in two or more populations, this is considered parallel evolution without considering the magnitude of change.

*Phenotypes*

The analysis of phenotypic traits is complicated by our ignorance about the evolution of phenotypes under neutrality. Although some models, such as Brownian motion [49], have been suggested, the general implicit consensus is to assume that phenotypic means do not change without directional selection or selection in response to a shift in trait optimum (e.g. [50,51]). Although this assumption is reasonable, in particular for traits under stabilizing selection [52], it is frequently requested that a significant change needs to occur in at least two replicate populations [53,54]. When phenotypes are measured for multiple individuals from the same population an analysis of variance can be used to measure a population × treatment interaction effects. Significant interactions indicate non-parallel selection responses [22,47].

## Data Availability

This article has no additional data.
